# A Comparative Analysis of the Status Anxiety Hypothesis of Socio-economic Inequalities in Health Based on 18,349 individuals in Four Countries and Five Cohort Studies

**DOI:** 10.1038/s41598-018-37440-7

**Published:** 2019-01-28

**Authors:** Richard Layte, Cathal McCrory, Cliona Ni Cheallaigh, Nollaig Bourke, Mika Kivimaki, Ana Isabel Ribeiro, Silvia Stringhini, Paolo Vineis

**Affiliations:** 1 The Department of Sociology, School of Social Sciences and Philosophy, Trinity College Dublin, Dublin, Ireland; 2 The Irish Longitudinal Study on Ageing (TILDA), Trinity College Dublin, Dublin, Ireland; 3 Centre for Medical Gerontology, Trinity College Dublin, Dublin, Ireland; 4 Institute of Epidemiology & Health, University College London, London, UK; 5 Departamento de Ciências da Saúde Pública e Forenses e Educação Médica, Faculdade de Medicina, Universidade do Porto, Porto, Portugal & EPIUnit, Instituto de Saúde Pública da Universidade do Porto, Porto, Portugal; 6 Institute of Social and Preventive Medicine, Lausanne University Hospital, Lausanne, Switzerland; 7 MRC-PHE Centre for Environment and Health, School of Public Health, Department of Epidemiology and Biostatistics, Imperial College London, London, UK

## Abstract

The status anxiety hypothesis proposes that systematic inflammation as a consequence of chronic psycho-social stress is a possible pathway linking socio-economic position (SEP) to premature ageing and is a possible explanation for cross-national variation in patterns of health and well-being. Harmonised data from the LIFEPATH consortium on 18,349 individuals aged 50 to 75 and 30,632 observations are used to measure variation in the association between inflammation measured as C-reactive protein and SEP across four countries (Britain, Ireland, Portugal and Switzerland) and five studies (ELSA, Whitehall II, TILDA, EPIPorto and SKIPOGH). Adjusting for population composition, mean concentrations of CRP are highest in Portugal, the country with the highest income inequality and lowest in Switzerland, a lower income inequality country. Across all of the studies, lower SEP groups have higher mean concentrations of CRP and, as predicted by the theory, absolute differentials between SEP groups reflect the pattern of societal income inequality. Adjustment for lifestyle indicators reduces SEP differentials by between 45% and 52% but cannot account for country variation in mean inflammation.

## Introduction

The ‘inverse health gradient’ between socioeconomic position (henceforce SEP), health and life expectancy is now a well-established fact across all societies where evidence has been collected^1^. Irrespective of the measure of SEP used (income, education, social class or status), socially and economically disadvantaged individuals have a shorter life expectancy and worse health during life^2^. The gradient in health emerges early in childhood and gets steeper with age^3,4^.

SEP determines the level of resources available to individuals and households, influencing biological function via the material environment, e.g. the location and quality of housing and exposure to pollutants, damp and microbial load^5^ as well as health behaviours^6^ but it is becoming increasingly clear that SEP may also shape biological health via neurological and hormonal pathways^7^. Under this paradigm, chronic psycho-social stress caused by economic strain^8^, uncertainty and lack of control^9^ or threats to social status and social threat^10–12^ are purported to cause SEP inequalities in health either directly or indirectly via adverse health behaviours. The direct pathway occurs through activation of glucocorticoid and adrenergic signalling pathways^13,14^ that, amongst other consequences, result in a chronic inflammatory response. There are now a number of studies which provide evidence of an association between SEP and systematic inflammation in adulthood^15–19^. A possible physiological process mediating the association between chronic psycho-stress and inflammation has been shown by Tawakol *et al*.^20^. Raised amygdala activation increases haemopoietic activity and arterial inflammation leading to an elevated risk of cardiovascular events. The same underlying process has been shown to contribute to the risk of other diseases such as type 2 diabetes^21^ and cancer^22^.

The emerging link between psycho-social stress and disease has also been put forward as an explanation for the pattern of cross-national variation in health and well-being^23–26^. Proponents of this hypothesis, known as the *status anxiety hypothesis*, argue that, irrespective of national wealth, all developed societies exhibit a gradient in levels of morbidity and mortality by SEP rather than a step-function between advantaged and disadvantaged. Absolute variation in morbidity and mortality across societies is more strongly correlated with level of income inequality than to gross domestic product per capita; among rich developed countries, differences in income and wealth matter less than how they are distributed across the population. Proponents of the status anxiety hypothesis put forward three linked propositions. First, that human social psychology was shaped by evolution which occurred almost entirely within hunter-gather societies with limited inequalities in material standard of living and social status^27^. Second, that income inequality is one measure of a society’s hierarchy of social status and third, that more inequality in the distribution of income and other scarce resources^28^, leads to increased psycho-social stress at the individual level at *all points in the SEP distribution* with consequences for health and well-being. If true, this hypothesis would imply that the differential between low and high SEP in status anxiety, psycho-social stress and inflammation will be positive with income inequality. Another implication is that *everyone* experiences higher psycho-social stress and inflammatory response in more unequal societies, not just those in the lowest SEP.

The extent of perceived *status anxiety* has been shown to vary significantly across European countries by SEP^28,29^ and more unequal countries (as measured by income inequality) have larger differentials in perceived status anxiety by SEP^29,30^ and worse mental health^30^. Moreover, more unequal countries have higher status anxiety overall. The highest SEP groups in more unequal societies have similar levels of status anxiety to the lowest SEP groups in more equal societies^29^. However, to date, there have been no studies comparing SEP differentials in inflammation across countries which vary by level of income inequality. If the status anxiety hypothesis is correct, higher income inequality should be associated with larger social differentials in inflammation between SEP and a higher mean level of inflammation. Alternative hypotheses for the association between income inequality and health have been proposed which would not implicate psycho-social stress and inflammation^30–32^ but evidence of social structuring of inflammation by SEP and country inequality would be supportive of the status anxiety hypothesis.

However, average levels of inflammation could also vary within and between societies as a function of health behaviours. Chronic psycho-social stress has been shown to promote behaviours with adverse effects on health such as smoking, poor diet and excess alcohol consumption^9^ and these variables have been shown to be a mediator between SEP and inflammation^16,17,19,33^.

**Figure 1:**
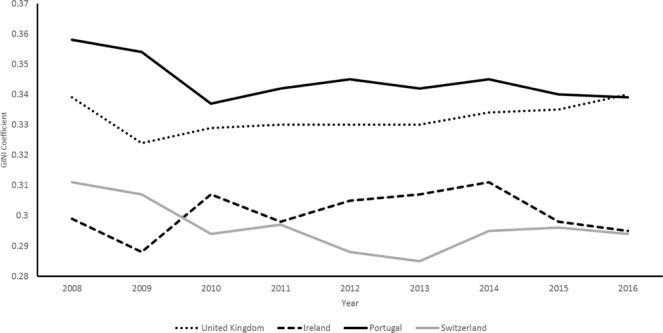
GINI Coefficient (Income Inequality) by Year and Country.

In this paper, we examine the prediction of the *status anxiety hypothesis* that countries with higher income inequality will have larger differentials in inflammation by SEP as well as higher average levels of inflammation in the population overall. Ideally, we would use individual level data with information on SEP, perceived status anxiety, inflammatory markers and lifestyle indicators for a large number of countries which differ in terms of income inequality, preferably measured with repeated observations over time. Unfortunately, such data are not available at present; instead, we draw on harmonised data on SEP (European Socio-economic Classification - EsEC), inflammation (C-reactive protein concentration – henceforth CRP) and lifestyle factors (smoking, body-mass index, diabetes & hypertension) from the LIFEPATH Project, an EC Horizon 2020 consortium, to carry out a more limited examination of the hypothesis in four European countries: Britain, Ireland, Portugal and Switzerland. Figure 1 shows that these countries have consistently had different levels of income inequality with Portugal having the highest average income inequality (measured using the GINI coefficient) at 34.5, followed by the United Kingdom at 33.2, Ireland at 30 and Switzerland at 29.6. These country differences in levels of income inequality are relatively muted in comparison to other nations. For example, the USA has a GINI coefficient of 45 compared to Sweden at 24.9. This would imply that our test of the status anxiety hypothesis is relatively conservative and more likely to produce a null-finding compared a test using countries which vary more in their levels of income inequality.

Data from The English Longitudinal Study of Ageing^34^ (ELSA) and the Whitehall II Study^35^ are used for the UK, The Irish Longitudinal Study of Ageing^36^ (TILDA) is used for Ireland, The Swiss Kidney Project on Genes and Hypertension^37^ (SKIPOGH) for Switzerland and the EPIPorto Study^38^ for Portugal.

## Results

Of the 34262 individuals who participated across the five studies, varying proportions contributed blood samples for the collection of CRP. This proportion varied from 39% in EPIPorto to 77% in ELSA. To provide comparative samples across studies, individuals aged between 50 and 75 were selected, reducing the sample to 19,018 individuals overall with at least one CRP sample. Given our focus on chronic inflammation, participants with CRP levels higher than 10 mg/L were excluded from the analysis sample as this could indicate the presence of an acute illness, reducing the sample to 18,349 individuals. Inclusion of multiple observations per person for some samples (Whitehall, SKIPOGH, ELSA) provides 30,632 observations overall. Table 1 provides the distribution of individual characteristics for the sample of individuals by study. The distribution of SEP indicators across countries has important implications for the distribution of lifestyle factors and the patterning of CRP. Three of the samples for analysis (SKIPOGH, TILDA and ELSA) have approximately equal proportions of men and women whereas the EPIPorto sample is 64% female and Whitehall II 69% male. EPIPorto has a higher proportion of the lowest social class (56%) and education groups (72%) compared to the other studies which may lead to higher overall levels of inflammation. The Whitehall II study population is more advantaged in its socio-economic profile. The distribution of lifestyle indicators vary widely across countries: 33% of the TILDA sample are measured as obese compared to just over 18% in Whitehall II and 17% in SKIPOGH. To ensure that the samples are compared on a common basis, sample weights are used to create equal sample distributions for each of the studies by sex, age group and SEP. Distributions of individuals by sex, age and SEP are thus identical across samples in analyses.

**Table 1:** Final Sample Characteristics by Study (of individuals).

	EPIPorto		Whitehall		SKIPOGH		TILDA		ELSA	
N Individuals	817		7,175		487		4,570		5,300	
N Observations	817		13,733		813		4,570		10,699	
	N	(%)	N	(%)	N	(%)	N	(%)	N	(%)
**Socioeconomic Position**										
**Most Recent Social Class**										
High Class	146	17.9	3,832	53.4	112	23.0	1,549	33.9	1,341	25.3
Med. Class	211	25.8	2,191	30.5	169	34.7	1,105	24.2	2,259	42.6
Low Class	460	56.3	1,152	16.1	206	42.3	1,916	41.9	1,700	32.1
**Highest Education**										
High Education	139	17.0	2,449	34.3	87	17.9	785	17.2	798	15.3
Med. Education	91	11.1	1,919	26.9	145	29.8	1,676	36.7	1,180	22.6
Low Education	587	71.9	2,764	38.8	255	52.4	2,109	46.2	3,243	62.1
**Demographics**										
**Sex**										
Men	295	36.1	4,974	69.3	221	45.4	2,148	47.0	2,445	46.1
Women	522	63.9	2,201	30.7	266	54.6	2,422	53.0	2,855	53.9
**Age Groups**										
Age 50–54	204	25.0	2,714	37.8	135	27.7	1,071	23.4	669	12.6
Age 55–59	185	22.6	2,409	33.6	102	20.9	1,124	24.6	1,493	28.2
Age 60–64	162	19.8	1,213	16.9	78	16.0	949	20.8	1,100	20.8
Age 65–69	142	17.4	662	9.2	95	19.5	789	17.3	1,076	20.3
Age 70–74	124	15.2	177	2.5	77	15.8	637	13.9	962	18.2
**Lifestyle Indicators**										
**Body Mass Index**										
Healthy Weight	227	27.9	2,463	35.8	211	43.3	1,006	22.1	1,309	26.3
Overweight	380	46.7	3,193	46.4	195	40.0	2,049	44.9	2,258	45.3
Obese	207	25.4	1,220	17.7	81	16.6	1,507	33.0	1,414	28.4
**Diabetes**										
No	723	88.5	6,761	95.3	471	96.7	4,284	93.7	4,313	94.2
Yes	94	11.5	337	4.8	16	3.3	286	6.3	268	5.9
**Tobacco Consumption**										
Never Smoker	531	65.2	3,237	47.7	209	42.9	2,081	45.5	2,208	41.7
Past Smoker	186	22.9	2,638	38.9	194	39.8	1,747	38.2	2,296	43.3
Current Smoker	97	11.9	913	13.5	84	17.3	742	16.2	794	15.0
**Hypertension**										
No	340	41.6	5,625	78.5	404	83.0	4,262	93.3	3,196	62.8
Yes	477	58.4	1,538	21.5	83	17.0	308	6.7	1,891	37.2

### Descriptive Differentials in CRP Concentration

Using weights to adjust for sample composition across studies, mean observed CRP concentration is significantly higher for low SEP compared to high SEP in all studies and across the age range, although the pattern is less distinct for SKIPOGH (Table 2), for the contrast between medium SEP and high and when education is the SEP measure. Being overweight and particularly being defined as obese, is associated with a significantly higher CRP than among those who are healthy weight. The absolute increase in CRP with obesity is higher than when the individual is overweight. The pattern of increases in CRP with indications for diabetes and hypertension are more mixed with consistent, significant differences in Whitehall II and ELSA but not in the other studies. Being a current daily smoker is associated with a significantly higher mean CRP, although not in EPIPorto. Women have higher mean CRP at all ages and across studies.

**Table 2:** Observed (Unweighted) Mean Deviation in CRP Concentration (mg/L) by Characteristic, Study and Age Group.

Characteristic	Study	Age Group				
		50–54	55–59	60–64	65–69	70–75
Social Class Low v Social Class High	EPIPorto	−0.01	**0.54**	**0.70**	**0.96**	**0.63**
	Whitehall	***0.58***	***0.62***	***0.71***	***0.60***	***1.02***
	SKIPOGH	0.02	***0.75***	−0.14	**0.55**	−0.18
	TILDA	***0.33***	***0.45***	**0.28**	**0.30**	***0.55***
	ELSA	***0.42***	***0.59***	***0.63***	***0.53***	***0.28***
Social Class Medium v Social Class High	EPIPorto	−0.24	**0.88**	−0.14	0.05	0.54
	Whitehall	***0.29***	***0.30***	***0.36***	***0.60***	***0.66***
	SKIPOGH	0.24	0.37	−0.20	***0.77***	0.11
	TILDA	**0.22**	0.14	0.12	−0.05	***0.69***
	ELSA	−0.16	***0.31***	**0.16**	***0.32***	0.09
Education Low v Highest Education High	EPIPorto	**−0.03**	0.36	***1.00***	***1.54***	**0.68**
	Whitehall	***0.26***	***0.33***	***0.36***	***0.38***	***0.50***
	SKIPOGH	***0.92***	**0.76**	0.29	0.37	0.01
	TILDA	**0.27**	***0.71***	***0.51***	0.08	***0.68***
	ELSA	***0.63***	***0.56***	***0.79***	***0.69***	***0.42***
Highest Education Medium v Highest Education High	EPIPorto	−0.83	***0.79***	0.67	0.60	1.01
	Whitehall	***0.12***	**0.11**	**0.15**	**0.23**	0.00
	SKIPOGH	**0.44**	−0.08	0.25	*0.38*	0.34
	TILDA	**0.25**	**0.21**	***0.42***	−0.10	**0.39**
	ELSA	***0.41***	***0.24***	***0.56***	***0.48***	0.23
BMI Overweight v Healthy Weight	EPIPorto	**0.49**	***0.56***	−0.03	0.07	0.38
	Whitehall	***0.56***	***0.65***	***0.66***	***0.66***	***0.63***
	SKIPOGH	***0.50***	***0.66***	0.39	0.36	***0.99***
	TILDA	***0.49***	***0.54***	0.15	***0.47***	0.05
	ELSA	***0.58***	***0.61***	***0.63***	***0.39***	***0.27***
BMI Obese v Healthy Weight	EPIPorto	***1.12***	***1.83***	**0.64**	*0.59*	***0.59***
	Whitehall	***1.64***	***1.62***	***1.63***	***1.63***	***1.56***
	SKIPOGH	***1.69***	***2.24***	***1.99***	***1.88***	***1.53***
	TILDA	***1.49***	***1.47***	***1.08***	***1.32***	***0.76***
	ELSA	***1.68***	***1.53***	***1.57***	***1.52***	***1.11***
Diabetes Indication v No Diabetes Ind.	EPIPorto	***1.07***	**0.61**	−0.24	−0.03	0.14
	Whitehall	***1.13***	***1.01***	***0.79***	***0.39***	***0.98***
	SKIPOGH	1.29	0.62	0.88	***1.78***	0.02
	TILDA	***1.06***	0.34	**0.40**	0.06	0.04
	ELSA	***0.84***	**0.35**	***1.23***	−0.07	−0.16
Hypertension Indicated v No Hypertension Indicated	EPIPorto	***0.69***	***0.84***	**0.59**	−0.20	0.22
	Whitehall	***0.37***	***0.44***	***0.38***	***0.35***	***0.43***
	SKIPOGH	0.22	−0.10	0.18	0.12	0.16
	TILDA	***0.61***	0.16	−0.14	−0.10	−0.29
	ELSA	0.09	***0.36***	***0.50***	***0.20***	***0.32***
Smoked but Quit	EPIPorto	**−0.54**	**−0.62**	−0.49	***−1.30***	**−0.73**
	Whitehall	0.07	0.04	**0.09**	−0.04	0.05
	SKIPOGH	−0.29	0.06	−0.25	**0.44**	0.11
	TILDA	**0.23**	−0.03	0.10	***0.38***	0.05
	ELSA	−0.12	−0.04	0.04	0.10	0.08
Daily Smoker	EPIPorto	0.18	**0.87**	0.41	−0.48	1.06
	Whitehall	***0.76***	***0.67***	***1.01***	***0.87***	***1.78***
	SKIPOGH	−0.22	**−0.54**	0.15	**0.77**	0.06
	TILDA	***0.61***	***0.53***	***0.75***	***0.67***	0.21
	ELSA EPIPorto	***0.69***	***0.60***	***0.85***	***0.84***	***0.49***
Female v Male		***0.62***	**0.46**	0.39	***0.89***	***1.04***
	Whitehall	***0.35***	***0.42***	***0.54***	***0.56***	***0.99***
	SKIPOGH	**0.56**	−0.09	0.15	0.10	0.34
	TILDA	***0.35***	***0.25***	0.12	−0.09	***0.33***
	ELSA	***0.27***	***0.18***	***0.24***	***0.20***	***0.33***

Key: Bold Alone - P < 0.05; *Italic Alone* - P < 0.01; *Bold & Italic*: P < 0.001.

### Predicted Country Differentials in CRP Concentration by Study

**Figure 2:**
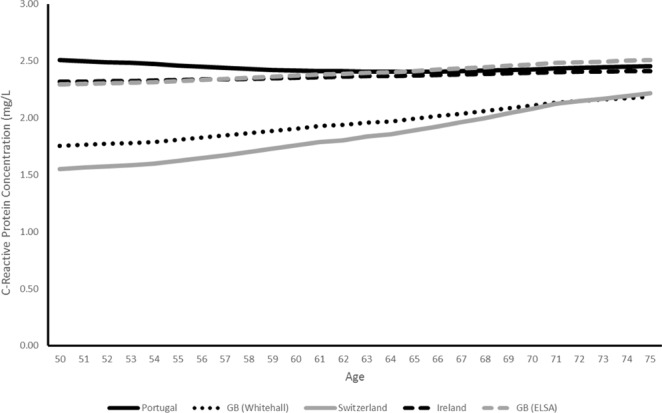
Predicted Overall CRP Concentrations by Country and Age Trajectory, (mg/L).

Using weights to adjust for sample composition across studies (Fig. 2), predicted mean concentrations of CRP are highest in EPIPorto (2.42 mg/L: 95% CI 2.24–2.59) and ELSA (2.42 mg/L: 95% CI 2.37–2.47) and lowest in SKIPOGH (1.87 mg/L: 95% CI 1.71–2.04) with the lower mean concentrations for Whitehall II and SKIPOGH significantly lower than those for EPIPorto and ELSA (P < 0.001). Between study variations in absolute mean CRP concentration falls with age and all mean differences in CRP between studies become insignificant by age 70. Confidence intervals for mean CRP concentration for EPIPorto, ELSA and TILDA overlap, but the pattern of study differences in mean CRP is broadly in line with the hypothesis that the country with the highest overall income inequality (Portugal/EPIPorto) will have higher average levels of inflammation compared to the country with the lowest (Switzerland/SKIPOGH).

### Predicted Absolute Differentials in CRP Concentration

Table 3, Panel A provides the estimated CRP concentrations (mg/L) by SEP obtained from the weighted generalized linear models with terms for age, sex, study and interactions of study, SEP and sex with polynomials of age. These confirm that across the studies, the low and middle SEP have significantly higher concentrations of CRP compared to the high SEP in all studies. Absolute differentials between low SEP and high SEP are largest in EPIPorto (0.52 mg/L: 95% CI 0.37–0.68) and lowest in SKIPOGH (0.27 mg/L: 95% CI 0.23–0.3).

**Table 3:** Predicted C-reactive (mg/L) levels by study and SEP.

	A: Unadjusted					B: Adjusted				
	Mean	95% CI		P Value	% Increase	Mean	95% CI		P Value	% Increase
**EPIPorto**										
SEP High	2.10	1.72	2.47	Ref.		1.43	1.18	1.67	Ref.	
SEP Medium	2.44	2.14	2.74	<0.001	16%	1.59	1.38	1.79	<0.001	11%
SEP Low	2.62	2.40	2.84	<0.001	25%	1.63	1.47	1.79	<0.001	14%
**Whitehall II**										
SEP High	1.72	1.58	1.86	Ref.		1.24	1.15	1.34	Ref.	
SEP Medium	2.11	2.01	2.22	<0.001	23%	1.42	1.33	1.50	0.055	14%
SEP Low	2.24	2.09	2.38	<0.001	30%	1.46	1.33	1.59	0.004	17%
**SKIPOGH**										
SEP High	1.64	1.37	1.90	Ref.		1.24	1.03	1.45	Ref.	
SEP Medium	1.91	1.63	2.18	<0.001	17%	1.37	1.19	1.56	<0.001	11%
SEP Low	1.90	1.67	2.14	<0.001	16%	1.29	1.13	1.45	<0.001	4%
**TILDA**										
SEP High	2.16	2.06	2.25	Ref.		1.40	1.32	1.48	Ref.	
SEP Medium	2.34	2.23	2.45	<0.001	9%	1.51	1.41	1.60	<0.001	8%
SEP Low	2.57	2.48	2.67	<0.001	19%	1.54	1.46	1.63	<0.001	10%
**ELSA**										
SEP High	2.17	2.07	2.27	Ref.		1.41	1.33	1.50	Ref.	
SEP Medium	2.32	2.24	2.39	<0.001	7%	1.49	1.41	1.56	<0.001	5%
SEP Low	2.62	2.52	2.71	<0.001	21%	1.59	1.50	1.68	<0.001	12%

Predicted values adjust for sex, age, age^2^ using study specific generalized linear models. Panel B adjusts additionally for smoking, indications of hypertension, BMI and indications of diabetes.

**Figure 3:**
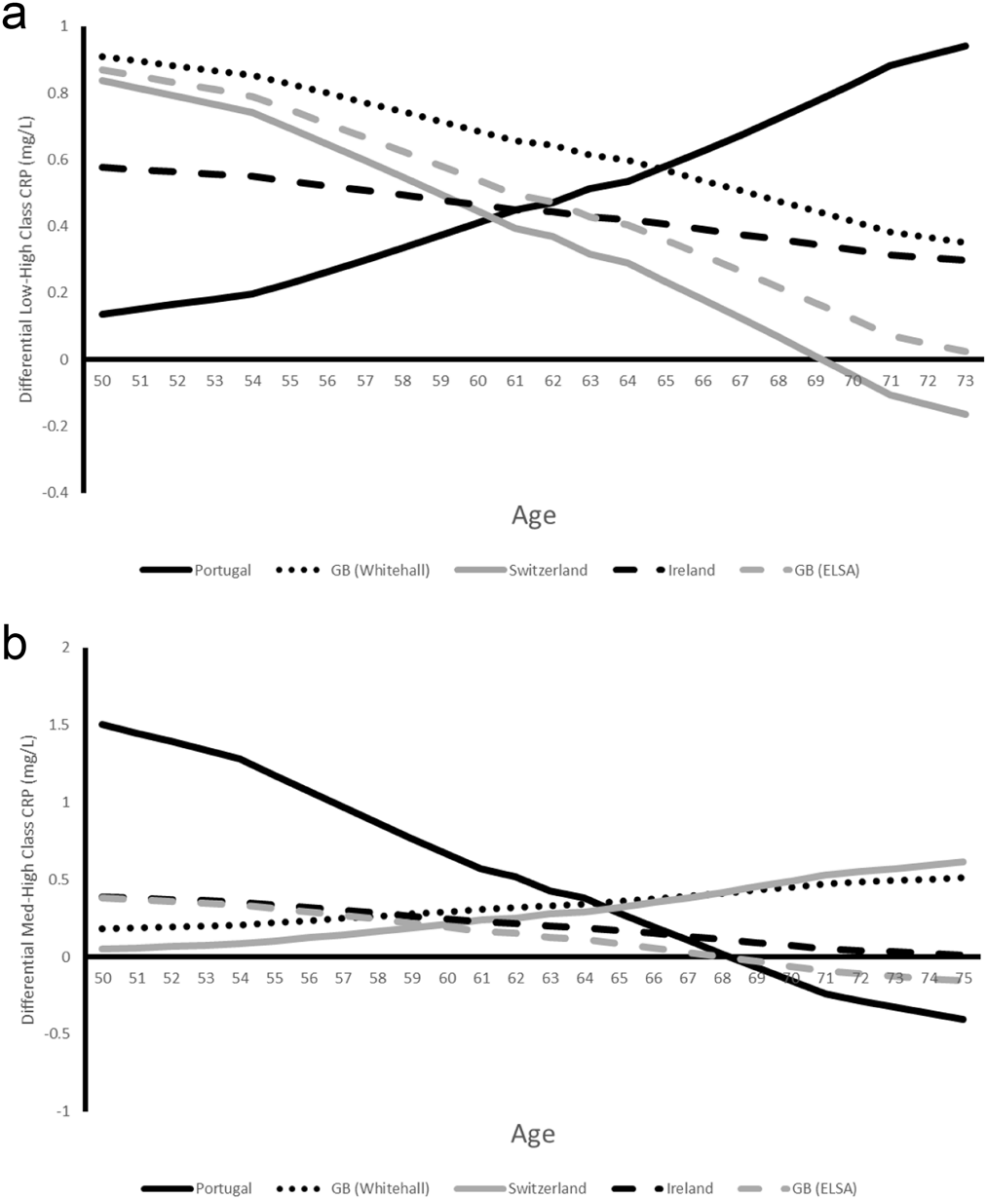
(**a**) Predicted Absolute Differentials (Low SEP – High SEP) in CRP Concentration (mg/L) by Country and Age. (**b**) Predicted Absolute Differentials (Middle SEP – High SEP) in CRP Concentration (mg/L) by Country and Age.

Predicted SEP differentials by age vary across studies (Fig. 3a,b). In all studies except EPIPorto, absolute differentials between low and high SEP groups fall with age and lowest in TILDA then SKIPOGH from age 60. The low-high SEP differential for EPIPorto on the other hand increases steeply with age from 0.14 mg/L at 50 (95% CI: 0 to 1.39) to 1 mg/L at 75 (95% CI: 0 to 2.76) although wide confidence intervals for the SEP differential in EPIPorto mean that the pattern could logically also be decreasing rather than increasing.

**Figure 4:**
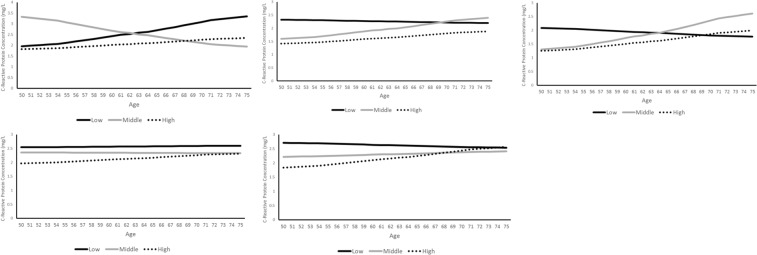
(**a**) Predicted CRP Concentration by Class and Age Trajectory, (mg/L) Portugal. (**b**) Predicted CRP Concentration by Class and Age Trajectory, (mg/L) GB (Whitehall II). (**c**) Predicted CRP Concentration by Class and Age Trajectory, (mg/L) Switzerland. (**d**) Predicted CRP Concentration by Class and Age Trajectory, (mg/L) Ireland. (**e**) Predicted CRP Concentration by Class and Age Trajectory, (mg/L) GB (ELSA).

Figure 4a–e show the predicted age trajectories of CRP by SEP for the five studies and illustrate why the SEP differential with age is different for EPIPorto. In that study, CRP rises for both low and high SEP, but the increase is steeper for the low SEP group.

### Relative SEP Differentials in CRP Concentration

**Figure 5:**
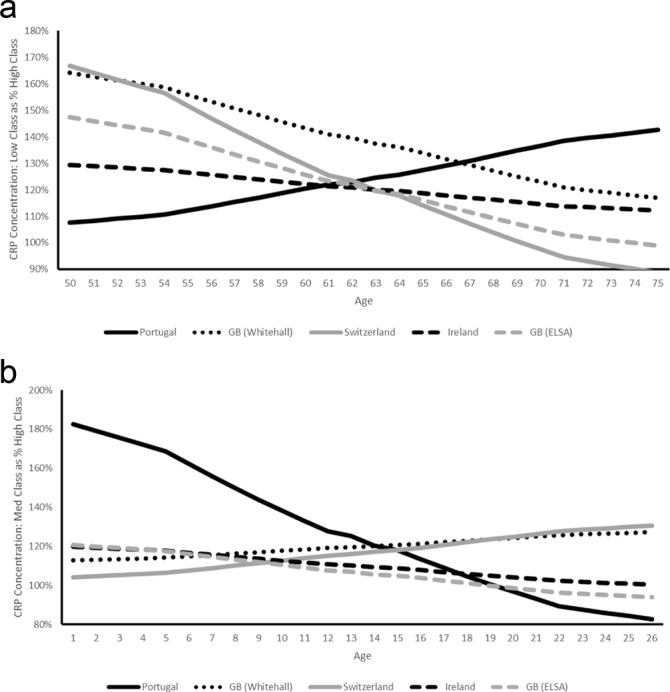
(**a**) Predicted Relative Differentials (Low SEP - High SEP) in CRP Concentration (mg/L) by Country and Age. (**b**) Predicted Relative Differentials (Middle SEP - High SEP) in CRP Concentration (mg/L) by Country and Age.

Relative SEP differentials (low/high SEP - Table 3, Panel A) are lowest in SKIPOGH (116%: 95% CI 111–118%) and highest in Whitehall II (130%: 95% CI 122–124%) and EPIPorto (125%: 95% CI 113–128%). The pattern of relative SEP differentials by age largely mirrors the pattern of absolute differentials by age (Fig. 5a,b) with decreases across all studies except EPIPorto, although, wide confidence intervals make interpretation problematic.

### SEP Differentials in CRP Concentration Adjusting for Lifestyle Factors

To examine the extent to which study and SEP differentials can be accounted for by differences in lifestyle behaviours across samples and groups, generalised linear models were estimated adjusting for the base model and lifestyle factors (Table 3, Panel B). With adjustment, both absolute and relative differentials in CRP between low, middle and high SEP groups fall but the differentials between low/middle and high SEP groups remains significant. The relative differential between low and high SEP groups after adjustment for lifestyle factors is lowest in SKIPOGH (104%: 95% CI 0% to 109%) and highest in Whitehall II (117%: 95% CI 114% to 116%).

**Figure 6:**
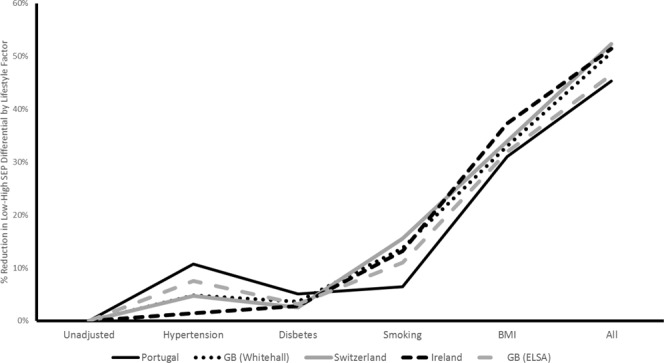
Predicted Reduction in Low/High SEP Differential by Adjustment of Lifestyle Factors by Country.

Figure 6 gives the reduction in relative differential between low and high SEP groups by the addition of lifestyle variables for each study. Single adjustment for BMI produces the largest reduction in differential (between 31% and 37% across studies). Adjusting for all of the lifestyle factors simultaneously decreases the low – high SEP differential by between 45% (EPIPorto) and 52% (SKIPOGH).

## Discussion

This paper provides new insight into the validity of the status anxiety hypothesis using biological data on inflammation measured as C-reactive protein. The hypothesis proposes that variation in income inequality between societies explains cross-national variation in differentials in health between SEP groups as well as at least some cross-national variation in population health. According to the hypothesis, societies which have larger social status differentials or which put more emphasis on social status will induce higher average levels of psycho-social stress with damaging biological consequences through activation of glucocorticoid and adrenergic signalling pathways^13,14^ that result in a chronic inflammatory response. Using data from five large cohort studies from four European countries we tested whether countries with higher income inequality will have higher average levels of inflammation and greater differentials in inflammation by SEP.

Overall, our findings support the predictions of the hypothesis: after constraining the samples to have the same age, sex and SEP distributions, mean concentrations of CRP are highest in Portugal, the country with the highest income inequality and lowest in Switzerland, the lower income inequality country, although country differences reduce with age and are not significant over the age of 70. Across all of the studies, lower SEP groups have a higher mean concentrations of CRP and, as predicted, absolute differentials between low SEP and high SEP are largest in Portugal (EPIPorto) and lowest in Switzerland (SKIPOGH).

Across all studies except EPIPorto, low SEP – high SEP differentials in inflammation are highest at age 50 and fall thereafter. The finding of decreasing differentials with age mirrors those of other studies internationally and support an ‘age as leveller’ process with the health status of surviving members of lower SEP groups converging with that of higher SEP groups. US and Finnish studies have previously found significant positive absolute differentials in younger age groups^39–41^ but diminishing or no differential for older age groups. Results from a recent British study are broadly consistent with this pattern although, differentials in CRP by income and education increased until age 60 before decreasing thereafter^19^.

Adjusting for the prevalence of different indicators of health behaviour reduces the country variation in relative SEP differentials in inflammation but does not fully explain the differential between low and high SEP groups. As found in previous studies^15,16,19,33^, adjustment for BMI across studies reduced differentials the most followed by adjustment for current and past smoking. Adjustment for all the lifestyle factors together reduced the low/high SEP differential by between 45% and 52%. The importance of BMI as a risk factor for raised levels of inflammation has been observed in numerous studies^15,16,19,33^ and reflects the active role of adipose tissue in endocrine function. Excess adipose tissue promotes inflammation due to its role as a store for macrophages^42^ and the pro-inflammatory effects of endogenous lipids^43,44^. A recent meta-analysis found that health behaviours mediated between 20% and 26% of the SEP differential in all-cause mortality, between 16 and 33% of the differential for cardiovascular disorders and between 17 and 29% of the differential for metabolic disorders^6^. Our results suggest a greater role for health behaviours in the development of inflammation, although it could be argued that our measures of health behaviours could be caused, at least in part, by the status anxiety process that we have been studying. If so, this would imply that we are statistically over-adjusting to a certain extent with these measures.

There are a number of potential limitations to this paper. First, the data available to us have limitations. Although we examine the predictions of the status anxiety hypothesis in terms of the impact of country income inequality on inflammation, we do not directly measure the mediating variable of status anxiety or psycho-social stress. Logically, inflammation at the individual level should vary with perceived status anxiety and the chronic stress response. There are no current data available, as far as we are aware, which contain survey measures of status anxiety alongside bio-markers of chronic stress (e.g cortisol) and inflammation.

A true test of the status anxiety hypothesis would include longitudinal data at the individual level on status anxiety and inflammation (as well as health behaviours and SEP) for a statistical sample of countries which vary widely in their levels of income inequality both within country over time and between countries in each time period. Such data would permit adjustment for country and individual fixed effects and confounding processes at the individual level whilst measuring the change in inflammation associated with a change in country level inequality. Unfortunately, internationally harmonised data on inflammatory markers is still very rare so our analysis is confined to four countries with a relatively narrow range in income inequality.

Second, the age range available for analysis was limited by the age range covered by the cohort studies available. Two of the five studies are older persons’ cohort studies (TILDA & ELSA) which include respondents aged 50+ thus setting the lower age limit. On the other hand, studies which include younger respondents have smaller numbers over the age of 75, limiting our observation period from age 50 to 75. It could be that the pattern of SEP differentials in SEP changes after age 75 although this is unlikely.

Third, this study used ESeC social class as the measure of SEP whereas other studies have used income and education measures. Past research has shown that income, education and social class have independent effects on health and mortality^2^, although all are correlated. Social class is often regarded as a measure of ‘permanent income’ as it tends to be correlated more highly with income averaged over a longer period of time than at any one point in time^2^ and so provides a less volatile measure than cross-sectional measures of income. Education, on the other hand, is an important predictor of income but its explanatory value varies widely with employment status, age and age cohort. Although CRP concentration varies over shorter periods of time, it is likely that chronic inflammatory response is a function of extended exposure to poor social and economic conditions thus social class is more highly correlated than income or education. Analyses of our data using highest education level as the SEP measure (available on request) show very similar patterns to the results using social class.

Fourth, this study focuses on CRP alone rather than analysing a range of inflammatory markers. This inevitably narrows the range of conclusions that can be drawn from the study. CRP is an acute phase protein, which is produced in response to innate pro-inflammatory cytokines including IL-6 and IL-1^45,46^. Low level elevations of CRP have been shown to correlate with childhood exposure to parental neglect^47^ and to be associated with increased risk of cardiovascular disease^48,49^ and cancer^50^. Our results are in line with those of other studies which have examined a wider set of inflammatory markers^19,39–41^. CRP is an endpoint of multiple pro-inflammatory pathways and does not capture anti-inflammatory pathways which are likely to be very important for the development of disease. CRP rises in acute inflammatory states in response to the production of innate immune inflammatory cytokines, but in homeostatic conditions, levels of CRP drop once inflammation is resolved^51^. Chronic elevation of CRP levels is therefore strongly indicative of a chronic pro-inflammatory state and persistently elevated CRP is a strong predictor of cardiovascular disease development, independent of traditional risk factors.

Fifth, the range of lifestyle indicators available on a harmonised basis was smaller than in other studies where detailed measures of diet and physical activity were available^19^. This means that we should be tentative about drawing firm conclusions about the extent to which we have adjusted for differences in lifestyles across studies and between social groups. It is likely that a wider or more detailed set of lifestyle indicators would increase the degree of attenuation produced and so reduce the residual differential between SEP groups.

Sixth, whilst our analyses suggest the health behaviours cannot account for the SEP differential in inflammation, this does not imply that the unexplained component should be attributed to solely to direct psycho-social processes. This paper could not exclude the role of a long list of other potential mediators between SEP and inflammation such as differentials in direct environmental exposures such as pollution, poor housing and microbial and parasitic load.

## Methods

### Data Sources

LIFEPATH is a Horizon 2020 funded project which aims to investigate the biological pathways which explain the differentials in premature ageing across different SEP. A consortium of 15 research teams harmonised data from 18 cohort studies from different countries which contained socio-economic, demographic, clinical and biological information. Detailed information on the LIFEPATH study and the harmonisation protocols used can be found elsewhere^52^. Five cohort studies (The English Longitudinal Study of Ageing^34^ (ELSA), The Irish Longitudinal Study of Ageing^36^ (TILDA), The Whitehall II Study^35^, The Swiss Kidney Project on Genes and Hypertension^37^ (SKIPOGH) and the EPIPorto Study^38^, all of which included data on the required variables: ESeC social class, CRP, age, sex, smoking, indications for diabetes, indications for hypertension and body mass index for individuals aged between 50 and 75. Overall, data on 18,349 individuals and 30,632 observations of CRP were available for analysis. Summary information on study design, years of recruitment and ethics review is available in Supplementary Table 1.

## Measures

### Inflammatory Markers

Inflammation was measured using CRP in mg/L using a high-sensitivity assay across all of the five studies. Multiple observations per person were available for Whitehall II, SKIPOGH, ELSA. CRP is extensively used as a biomarker clinically and in epidemiologic studies to define systemic inflammation.

### Socio-economic Position

Both individual social class and highest educational level were harmonised across the five studies. Social class was harmonised using the European Socio-Economic Classification (ESeC) which measures the individuals employment position within labour markets and production units based on a ten group classification^53^. For this paper, these ten groups have been collapsed to three groups: professional and managerial class; intermediate class; manual working class referred to as low, medium and high SEP. Educational qualifications across the four countries have been harmonised using the International Standard Classification of Education (ISCED 2011) and collapsed into three groups: lower secondary education or less; upper secondary education; third level education.

### Body Mass Index

Data from measured heights and weights was used to calculate body mass index using weight(kg)/height(m^2^) which was divided into underweight (<18.5), healthy (18.5–24.9), overweight (25–29.9) and obese (30+).

### Smoking

Current and past smoking behaviour was divided into three categories: never smoked (1), past smoker (2) and current smoker (3).

### Diabetes

Information on reported diagnosis by a clinician, reported diabetes medications and HbA1c measures were combined to produce a measure of diabetes. Respondents with a previous diagnosis, or who were currently prescribed medications of Anatomic Therapeutic Classification (ATC) codes ‘A10A’ for insulin and ‘A10B’ for oral ant-glycaemic medications or with a HbA1c of >=6.5% were defined as diabetic.

### Hypertension

Hypertension was defined as having an SBP >=140 mmHg and DBP >=90 mmHg or reported diagnosis by a clinician of high blood pressure or current use of any antihypertensive medication.

### Gender and Age

Age and gender are used to adjust for demographic composition. Age is used in categorical form in descriptive tables (50–54, 55–59, 60–64, 65–69, 70–75) and in continuous form in statistical modelling.

### Statistical analyses

Observed differentials in mean CRP by SEP, demographic, socio-economic and lifestyle indicator by age group and study were computed and statistical differences identified. Given previous research, overall mean concentrations of CRP by study and SEP will be determined by the sex, age and SEP distribution of the data and Table 1 provides evidence that these vary widely across the samples. To ensure that results do not reflect the composition of the samples, sample weights were designed which force each sample to replicate the sample composition of the ELSA sample by sex, age group and social class group. *Sample composition is therefore identical across studies* so variation between samples in CRP concentration cannot reflect variation in the sex, age or SEP composition of the sample.

CRP has a strong positive skew in all studies which makes it unsuitable for analysis using ordinary least squares regression. Instead, generalised linear models were employed which allow the conditional variance and link functions to be chosen which provide the best fit for the data. The modified Park test was used to select the former and Hosmer-Lemeshow test used to select the latter. Tests showed that the CRP distributions across all five studies was best modelled using Poisson GLM with log link. Individuals within the sample had a minimum of one and a maximum of three observations of CRP. The pooled sample across five studies contains at least one CRP observation on 19,018 individuals. Only individuals with at least one non-missing observation of CRP were retained for analysis but across study waves 30,632 observations were available for analysis as some individuals have multiple observations. Robust standard errors are used to adjust for the within individual correlation with observations. Missing data on all predictor variables was imputed using chained-equations implemented in STATA 15.

‘Base Models’ were estimated with terms for sex, age, study and SEP (measured as ESeC social class). To allow for flexible associations of age with CRP, different age polynomials were tested with quadratic and cubic age polynomial terms providing the best statistical fit based on reduction in log-likelihood. To allow for variation in the association between age and CRP across studies, interactions between study and both age, age^2^ and age^3^ were estimated. A second model tested for interactions between study, SEP, age and age polynomials to establish whether the SEP gradient varied across studies. In a third step, models were estimated to test whether SEP gradients in CRP could be explained by lifestyle variables. Measures for smoking, BMI, diabetes and hypertension were added separately and together to establish their individual and joint attenuating effects on the SEP/CRP association. Pooled models including both sexes were estimated but Interactions between gender and age were included to account for gender variation in the association between age and CRP.

### Ethical and regulatory approval

All of the data used in this paper were collected with ethical approval from the research ethics body of the institution involved. Details of the ethics body for each study are provided in Supplementary Table 1. Ethics review in each instance included scrutiny of the protocols and procedures followed for the collection of the biological samples and physical measures used in each study. The authors confirm that each study was carried out with the full informed consent of all subjects, that only adults aged 18 plus were included and that all human tissue was collected in accordance with relevant guidelines and regulations.

## Supplementary information

Supplementary Material
