# Supplementary material for: A Comparative Analysis of the Status Anxiety Hypothesis of Socio-economic Inequalities in Health Based on 18,349 individuals in Four Countries and Five Cohort Studies
Source: Sci Rep. 2019 Jan 28;9:796. doi: 10.1038/s41598-018-37440-7 (PMC6349896; doi:10.1038/s41598-018-37440-7)

A Comparative Analysis of the Status Anxiety Hypothesis of Socio-economic Inequalities in Health Based on 18,349 individuals in Four Countries and Five Cohort Studies

^1^Richard Layte, ^2^Cathal McCrory, ^3^Cliona Ni Cheallaigh, ^3^Nollaig Bourke, ^4^Mika Kivimaki, ^5^Ana Isabel Ribeiro, ^6^Silvia Stringhini and ^7^Paolo Vineis

^1^The Department of Sociology, School of Social Sciences and Philosophy, Trinity College Dublin, Ireland

^2^The Irish Longitudinal Study on Ageing (TILDA), Trinity College Dublin, Ireland

^3.^Centre for Medical Gerontology, Trinity College Dublin.

^4^Institute of Epidemiology & Health, University College London, London UK

^5^ Departamento de Ciências da Saúde Pública e Forenses e Educação Médica, Faculdade de Medicina, Universidade do Porto, Porto, Portugal & EPIUnit, Instituto de Saúde Pública da Universidade do Porto, Porto, Portugal.

^6^ Institute of Social and Preventive Medicine, Lausanne University Hospital, Lausanne, Switzerland

^7^ MRC-PHE Centre for Environment and Health, School of Public Health, Department of Epidemiology and Biostatistics, Imperial College London

Supplementary Table 1 : Study Design

|  | **Design** | **Sample Method and Size** | **Date of recruitment** | **Data collection** | **CRP measure** | **Period of first CRP measure used in the study** | **Ethics Approval** |
| --- | --- | --- | --- | --- | --- | --- | --- |
| **EpiPorto** | Prospective population cohort | 2485 individuals aged 18+ sampled using random-digit dialling of landlines | 1999 - 2003 | Participants completed a questionnaire on social, demographic and health characteristics including previous diagnoses of diabetes and hypertension and current and previous smoking behaviour. Respondents were invited to a study centre for collection of objective measures including blood and urine samples. | HS-CRP (mg/L) ssessed using particle enhanced  immunonephelometry using a Behring auto-analyser, Nephelometer  II, BN II (Dade Behring Marburg GMBH, Germany). | 1999 - 2003 | The Ethics Committee of the Hospital de São João approved the study protocol |
| **TILDA** | Irish Longitudinal Study of Ageing | 8175 individuals  Aged 50+ sampled from the GEO-Directory | 2009 - 2011 | Participants completed a personal interview and self-administered questionnaire and were then invited to attend one of two test centres for the collection of physical samples and bio-medical measures. | HS-CRP was measured in non-fasting blood plasma using a third generation particle enhanced immunoturbidimetric assay (Roche Diagnostics, UK). CRP concentrations were measured on a Roche Cobas 701 analyzer (Roche/Hitachi, UK). | 2009/2011 | The Research Ethics Committee of Trinity College Dublin. |
| **SKIPOGH** | The Swiss Kidney Project on Genes and Hypertension | 1189 individuals aged 18+ sampled using random-digit dialling of landlines | 2009 - 2013 | Participants completed a self-administered questionnaire at home on social, demographic and health behaviours and then received a home visit following an overnight fast at which blood and urine samples were collected and respondent height, weight and blood pressure (BP) were taken. | HS-CRP (mg/L) was assessed by immunoassay and latex HS (Roche Diagnostics, CH) | At baseline, between 2009 - 2013 | Human Research Ethics Committees for Lausanne University Hospital and University of Lausanne; the Ethics Committee for the Research on Human Beings, Geneva University Hospitals ; Ethics Committee of the Canton of Bern |
| **Whitehall II** | Prospective occupational cohort | 10,314 individuals aged 35 to 55 sampled from civil service records | From 1985 (12 waves) | Clinical examination and a self-administered questionnaire containing sections on demographic characteristics, health, lifestyle factors, work characteristics, social support, and life events. | CRP was measured using a high-sensitivity immunonephelometric assay in a BN ProSpec nephelometer (Dade Behring, Milton Keynes, Bucks, UK). | In the third wave, between 1991 - 1994 | The University College London ethics committee approved this study |
| **ELSA** | English longitudinal study of aging | 11391 individuals aged 50+ from Health Survey for England | Recruited from Health Survey for England in 1998, 1999, 2001 | Face-to-face interview (a computer-assisted personal interview followed by a self-completion questionnaire) every two years of the study and a nurse assessment every four years (measurements of physical function, anthropometric measurements and collection of blood samples). | CRP was measured using the N Latex CRP mono immunoassay on the Behring Nephelometer II analyzer. | 2004, 2008 and 2012 | Ethical consent was obtained for all waves and components of ELSA, according to the ethical approval system in operation at the time. |

Appendix Figure 1: Absolute Differentials (Low SEP – High SEP) in CRP Concentration (mg/L) by Study and Age – Men


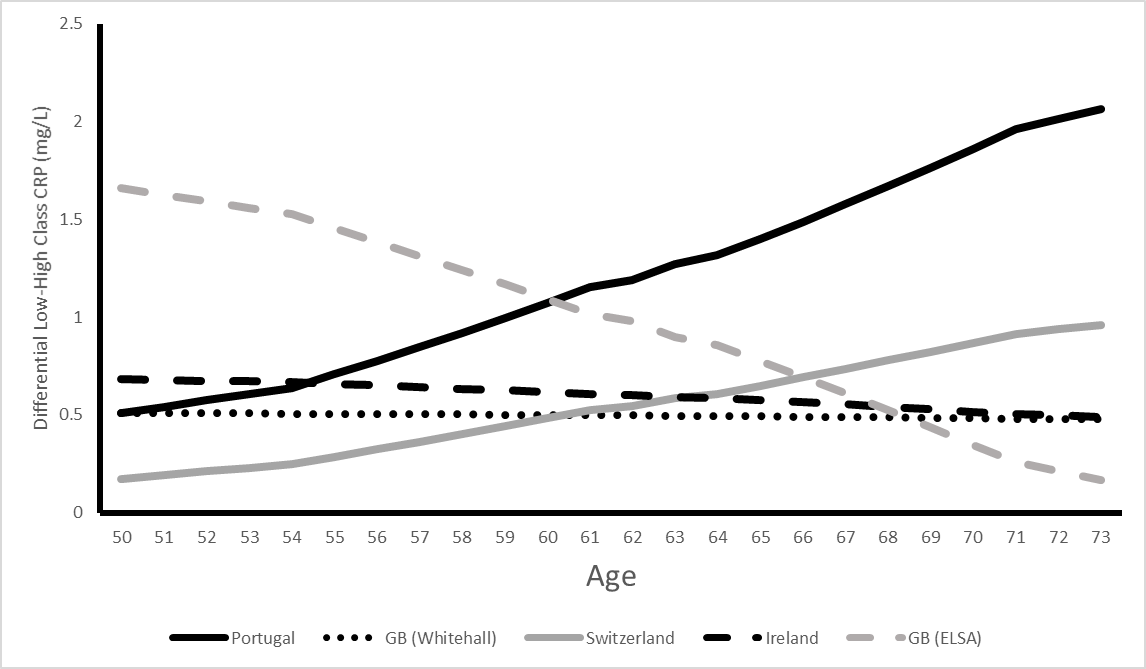


Appendix Figure 2: Absolute Differentials (Low SEP – High SEP) in CRP Concentration (mg/L) by Study and Age - Women


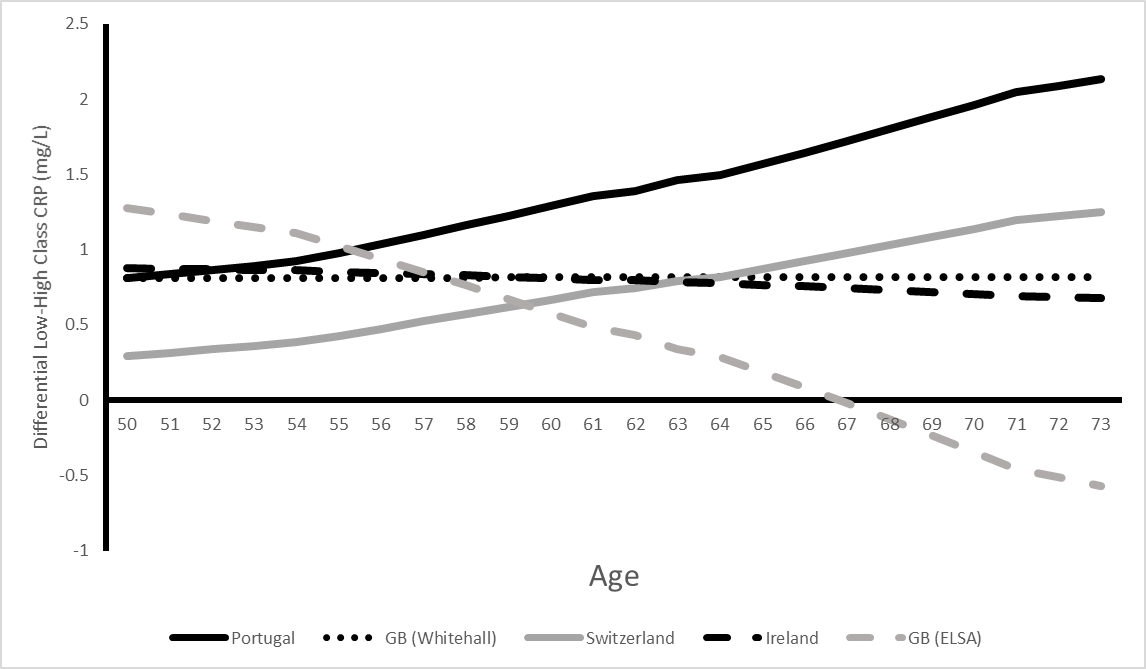

Supplement: Supplementary file 1 — Supplementary Material [file 41598_2018_37440_MOESM1_ESM.docx]
